# Label-free Quantitative Proteomic Analysis of Cerebrospinal Fluid and Serum in Patients With Relapse-Remitting Multiple Sclerosis

**DOI:** 10.3389/fgene.2022.892491

**Published:** 2022-04-27

**Authors:** Haijie Liu, Ziwen Wang, He Li, Meijie Li, Bo Han, Yuan Qi, Huailu Wang, Juan Gao

**Affiliations:** ^1^ Department of Neurology, Xuanwu Hospital, Capital Medical University, Beijing, China; ^2^ Department of Neurology, Baoding No. 1 Central Hospital, Baoding, China; ^3^ Department of Automation, College of Information Science and Engineering, Tianjin Tianshi College, Tianjin, China

**Keywords:** biomarker, cerebrospinal fluid, diagnosis, label free proteomics, relapse-remitting multiple sclerosis, serum

## Abstract

**Background:** The lack of effective serum and cerebrospinal fluid (CSF) biomarkers remains a barrier to early diagnosis and treatment of multiple sclerosis (MS). The study is to identify the diagnostic biomarkers of serum and CSF in patients who suffered MS.

**Methods:** At first, we performed differential analysis of CSF and serum proteomics on control and relapse-remitting multiple sclerosis (RRMS) patients. Secondly, CSF and serum’s differential proteins were compared, in order to identify the significative proteins. Finally, Kyoto Encyclopedia of Genes and Genomes (KEGG) and Gene Ontology (GO) analysis were performed on the differential proteins in serum and CSF respectively to clarify their common biological functions and pathways.

**Results:** At the first step, in CSF, 73 proteins were significantly differentially expressed in the RRMS set compared with the controls. In serum, 22 proteins were differentially expressed. Secondly, we found MMP2 C8G and CFH were the same high expression trend in CSF and serum. Finally, we found the differential proteins in serum and CSF are mostly participated in biological processes: immuno-inflammatory response, neuronal development, cell adhesion and signaling.

**Conclusion:** MMP2, C8G and CFH may participate in the pathogenesis of RRMS, which are the potential diagnostic biomarkers of the disease.

## 1 Introduction

Multiple sclerosis (MS) is a chronic immune-mediated inflammatory demyelinating disorder disease of the central nervous system. Although the mortality rate is not high, many people with MS develop into irreversible disability, which severely reduces their quality of life. Relapse-remitting multiple sclerosis (RRMS) is the most common form of MS. 80–85% of patients with MS initially show the course of relapsing remitting, which is characterized by an acute exacerbation of neurological symptoms and followed by complete or incomplete recovery. Therefore, early and accurate diagnosis of MS is vital to effective treatment and prevention of disease progression. Nowadays, the diagnosis of MS mainly depends on the clinic, which is based on the temporal and spatial multiplicity of central nervous system lesions. However, specific laboratory indicators of MS has not been found (Thompson et al., 2018). Different from neuromyelitis optica having the sensitive and highly specific serum marker (Antibodies to aquaporin-4, AQP4-Ab), the lack of effective biomarkers is an issue of MS diagnosis and treatment.

In a recent review of 24 studies on MS proteomics, more than 200 biomarkers of proteins were present in CSF, blood, urine, and brain tissue of patients with MS (Singh et al., 2019). A recent study used quantitative proteomics methods to analyze differences in CSF proteins between control and RRMS patients, chitinase 3-like proteins 1 (CHI3L1) and 2 (CHI3L2) are highly expressed in the brains of patients with MS (Hinsinger et al., 2015). Subsequently, candidate biomarkers in CSF and serum of MS were validated by enzyme linked immunosorbent assay (ELISA), confirming that CSF and serum’s CHI3L1 CHI3L2 levels were associated with the disease stage (Hinsinger et al., 2015). In addition, CSF proteins associated with the progression of MS were found to be involved in the immune response, blood coagulation, cell proliferation and cell adhesion (Füvesi et al., 2012). A study of MS patients in the United States showed significant differentially expressed in the proteins involved in the complement and coagulation pathways and lipid transport in the serum of MS patients compared with healthy controls (Wallin et al., 2015). In recent years, CSF is the target of most MS biomarker studies. Only a few studies have identified blood biomarkers, and even fewer focused on blood and CSF together. The only recent study of MS biomarkers in both CSF and blood was performed on European people. This study compared two separate cohorts of cases with healthy controls and patients with other neurological disorders (Huang et al., 2020). Using highly sensitive proteomic immunoassay, OSM HGF was identified as a proteins biomarker in MS plasma and IL-12B CD5 CCL3 CXCL9 as protein biomarkers in CSF (Huang et al., 2020). However, the study of biomarkers of combination on CSF and blood has not been performed on Asian people.

Therefore, as to extend the study to Asian people, we performed proteomic studies on CSF and serum of the same group of subjects simultaneously, and obtained the protein and biological function of CSF and serum that combined to cause RRMS, making certain contributions to the study of the pathological process and pathogenesis of RRMS. The results of the study will provide clues for further study on the validation of biomarkers in years to come.

## 2 Materials and Methods

### 2.1 Study Participants

The experimental protocol was approved by the Ethics Committee of Xuanwu Hospital Capital Medical University (reference no. LYS2021017), and conforms to the recommendations of Helsinki Declaration and subsequent revisions or corresponding ethical standards. All participants signed written informed consent after being informed of the detailed description of the entire study. CSF and serum from five patients with RRMS and five patients with oother non-inflammatory neurological diseases (ONIND) were included for proteomic studies. Peripheral blood from eight RRMS patients was used for genomic studies. The diagnostic criteria for RRMS patients is McDonald’s diagnostic criteria (Thompson et al., 2018). Spatiotemporal multiple is a major feature of these patients with MS, in which multiple lesions are found in the central nervous system and recurred over time. The diagnosis of MS was reconfirmed by measuring oligoclonal bands in peripheral blood and CSF. The clinical trial was conducted at Xuanwu Hospital Capital Medical University. Patients with RRMS and ONIND were recruited from the Department of Neurology. In this study, blood and CSF samples were collected before the treatment such as the hormone therapy and immunoglobulin therapy.

### 2.2 The Peripheral Blood and CSF Sample Collection

First, the whole blood of each sample was collected and placed in the corresponding sterile centrifuge tube, and then stand at 37 °C for 1 h for solidification stratification. Second, the whole blood was centrifuged at 2,000 rpm for 10 min at 4°C. Third, we separate at least 200 μL supernatant from each tube into the corresponding 1.5 ml sterile centrifuge tube, and keep it frozen at -80°C quickly for further study.

All the CSF samples were centrifuged at 1,500 rpm for 10 min at 4°C within 30 min. The supernatant was collected and stored at -80°C. To avoid protein degradation, no samples were repeatedly frozen and thawed.

### 2.3 Protein Extraction and Digest

Human serums, mixed in each group, were prepared by centrifugation at 4,000 g for 10 min and removed interfering high-abundance proteins of serum by the Agilent Multiple Affinity Removal Column (Multiple Affinity Removal Column, 4.6 mm × 50 mm; Agilent Technologies, Palo Alto, CA) to extend the dynamic range of Liquid Chromatography-Tandem Mass Spectrometry (LC-MS/MS) and electrophoretic analysis. Low-abundance proteins obtained were further processed: centrifuged, desalted, concentrated, freeze dried, and reconstituted in lysis buffer (containing 8M urea buffer and 150 mM Tris HCl, pH 8.0). Subsequently, the Bradford assay was used to determine the concentration of various proteins in each sample. After protein quantification, 60 μg protein solution was put into a centrifuge tube, and dithiothreitol (DTT, 5μL, 1 mol/L) solution was added to shake well for 1 h at 37°C. The samples were diluted with iodoacetamide (IAA, 20μL, 1 mol/L) in darkness for 60 min, and digested by trypsin for 12 h at 37°C (Lee et al., 2015). Finally, the digested supernatant fractions were stored at −80°C until LC-MS/MS analysis. The extraction and digestion of proteins from human CSF follow the same procedure as serum.

### 2.4 Liquid Chromatography-Tandem Mass Spectrometry (LC-MS/MS) Analysis

The polypeptide mixture of each sample was identified by nano high-performance liquid chromatography and mass spectrometer. First, the samples were desalted and separated by Agilent 1,100 quaternary HPLC (Agilent, EASYnLC1000, United States). RP trap column (Thermo EASYcolumn SC200, 150 μm × 100 mm) was used to desalinate the samples, and C18 reverse-phase column (Thermo EASY-column SC001 traps, 150 μm × 20 mm) was used to separate the samples. In this system, mobile phase A was 0.1% formic acid in acetonitrile (2% acetonitrile), and mobile phase B was 0.1% formic acid in acetonitrile (84% acetonitrile). The analytical separation was run at a flow rate of 400 nL/min by using a linear gradient of phase B as follows: 0–45% for 100 min, 45–100% for 8 min, and 100% for 12 min. Second, Agilent 1,100 quaternary HPLC (Agilent, EASYnLC1000, United States) was used to identify the samples by mass spectrometry. In order to reduce the probability of unnecessary experimental errors caused by technical errors, we independently repeated all experimental steps three times (Lee et al., 2015).

### 2.5 Bioinformatics Analysis

The identified protein fragment profiles were retrieved from the human database in Mascot 2.1 program (Matrix science) with errors of 6ppm or 20ppm. Peptide false discovery rate (FDR) ≤ 0.01.

First, We analyzed the biological functions of the differentially expressed proteins, and the biological functions were divided into the cellular component (CC), molecular function (MF) and biological process (BP), according to the standard of gene ontology (GO) (http://www.geneontology.org, GO) (Wu et al., 2016) (Zhang et al., 2014). Second, GO enrichment analysis: we put all the differentially expressed proteins to the GO database of each term mapping and calculate the number of protein each term. Hypergeometric tests were then applied to find the GO items that were significantly enriched in the differential proteins compared to all protein backgrounds. The calculation formula is:

$$P=1-\sum_{i=0}^{m-1}\frac{\left(\begin{array}{c} M\\ i \end{array}\right)\left(\begin{array}{cc} N & -M\\ n & -i \end{array}\right)}{\left(\begin{array}{c} N\\ n \end{array}\right)}$$

“N” is the number of proteins with GO annotation information in all proteins. “n” is the number of differential proteins in “N”, “M” is the number of proteins annotated with a certain GO entry in all proteins, “m” is the number of differential proteins annotated with a certain GO entry. The threshold is *p*-value ≤ 0.05.

Using the Kyoto Encyclopedia of Genes and Genome (KEGG) database (http://www.genome.jp/kegg/pathway.html) signalling pathway analysis was carried out on the significant differentially expressed proteins. And using the default database as the background, using hypergeometric distribution, enrichment analysis was carried out on the pathway (Kanehisa et al., 2004).

### 2.6 Whole Genome Exon Sequencing

Genomic DNA was fragmented using NEBNext dsDNA Fragmentase (NEB, Ipswich, MA, United States), followed by repair, dAtailed addition, to the end of the DNA. Biotinylated RNA library baits and magnetic beads were mixed with the barcoded library for targeted regions selection using the SureSelect Human All Exon V6 Kit (Agilent Technologies, Palo Alto, Calif.). The captured sequences were further amplified for 150bp paired-end sequencing in Illumina X-ten system (Illumina, San Diego, CA, United States) (Peng et al., 2021a). To identify SNPs and InDels, the Burrows-Wheeler Aligner (BWA) (Li and Durbin, 2009) was used to align the clean reads from each sample against the reference genome. SNPs and InDels were filtered using GATK’s Variant Filtration with proper standards (-Window 4, -filter “QD < 2.0 || FS > 60.0 || MQ < 40.0 ″, -G_filter “GQ < 20″) and those exhibiting segregation distortion or sequencing errors were discarded.

## 3 Results

In the study, we investigate the CSF and serum proteome from patients who suffered RRMS (n = 5) and ONIND (n = 5) respectively, using label-free quantitative proteomics methods, to identify biomarkers that can be used as potential serum and CSF candidates for RRMS. In addition, whole-genome exon sequencing was performed in eight patients with RRMS to compare with the reference genome to explain the association between gene mutation and disease.

### 3.1 Characteristics of the Study Population

To conduct serum and CSF proteomics studies in RRMS, we compared sex, age, and total CSF protein concentrations in five controls and five RRMS patients (Table 1). There is no significant differences in gender and age between the two groups, and the CSF protein concentration in the RRMS group was significantly higher than which in the control group. CSF immune indices were significantly different between the control group and the RRMS group: compared with the control group, RRMS patients had higher white blood cells counts in CSF, 24-h lgG index, albumin quotient, and oligoclonal bands appear (Table 1). General information about eight RRMS patients who participated in whole genome sequencing can be seen in Table 1.

**TABLE 1:** Demographic characteristic of the participants.

Characteristic	Proteomics Research		Genomics Research
	RRMS	ONIND	RRMS
N	5	5	8
N,CSF:serum	5:5	5:5	-
Age at onset, years	40.4 ± 6.6	47.0 ± 8.6	39.4 ± 7.3
Male:female ratio	1:0.67	1:0.67	1:1
CSF protein level (mg/L)	34.12 ± 7.31	29.66 ± 5.70	37 ± 7.87
Albumin quotient	0.14 ± 0.04	0.09 ± 0.02	0.12 ± 0.03
Positive OCBs (%)	0.5 ± 0.45	0	0.5 ± 0.43
IgG 24 h (mg/24 h)	12.14 ± 6.69	0.96 ± 1.17	11.50 ± 5.88
CSF WBCs count (*10^6^/L)	7.2 ± 1.94	1 ± 0.63	5.25 ± 2.90
Serum CRP (mg/L)	3.68 ± 3.10	3 ± 3.52	4.20 ± 2.58
Disease duration, years	4.34 ± 3.38	—	3.81 ± 4.02

RRMS: relapsing–remitting multiple sclerosis; ONIND: other non-inflammatory neurological diseases; CSF: cerebral spinal fluid; OCBs: oligoclonal bands; IgG: immunoglobulin G; WBCs: white blood cells; CRP: C-reactive protein. The value is represented by the mean ± SD.

### 3.2 Label-free Quantitative Proteomics Analysis

Label-free quantitative proteomics was used to analyze the proteins expression of the two groups (RRMS and ONIND). A total of 972 proteins were identified and quantitatively analyzed in CSF by the identification of one or more unique peptides. A total of 594 proteins were quantified in serum using the same method. Differential expressed proteins were defined as the fold change greater than 1.20 or less than 0.83 in relative abundance and a *p*-value < 0.05. Based on these criteria, there were 73 differentially expressed proteins from CSF samples between the RRMS group and ONIND group, and 22 differentially expressed proteins from serum samples (Supplementary Table S1; Supplementary Table S3). In the comparison between the RRMS group and ONIND group from CSF samples, 35 proteins were up-regulated (>1.2-fold) and 38 proteins were down-regulated (<0.83-fold) in the RRMS group. There were 14 up-regulated proteins (>1.2-fold) and eight down-regulated proteins (<0.83-fold) from serum samples. Compared with the ONIND group, three overlapping proteins were significantly differentially expressed in CSF and serum of the RRMS group (Table 2). C8G CFH and DSG2 were highly expressed in the CSF and serum.

**TABLE 2:** Three differentially expressed proteins common in CSF and serum (Fold change = 1.2).

Gene	Protein Name	Uniprot	CSF		Serum	
			Ratio	*p*-value	Ratio	*p*-value
MMP2	72 kDa type IV collagenase	P08253	1.3061	0.0153	1.4986	0.0340
CFH	Complement factor H	P08603	1.1395	0.0291	3.0811	0.0126
C8G	Complement component C8 gamma chain	P07360	1.5666	0.0275	1.3264	0.0238

**FIGURE 1:**
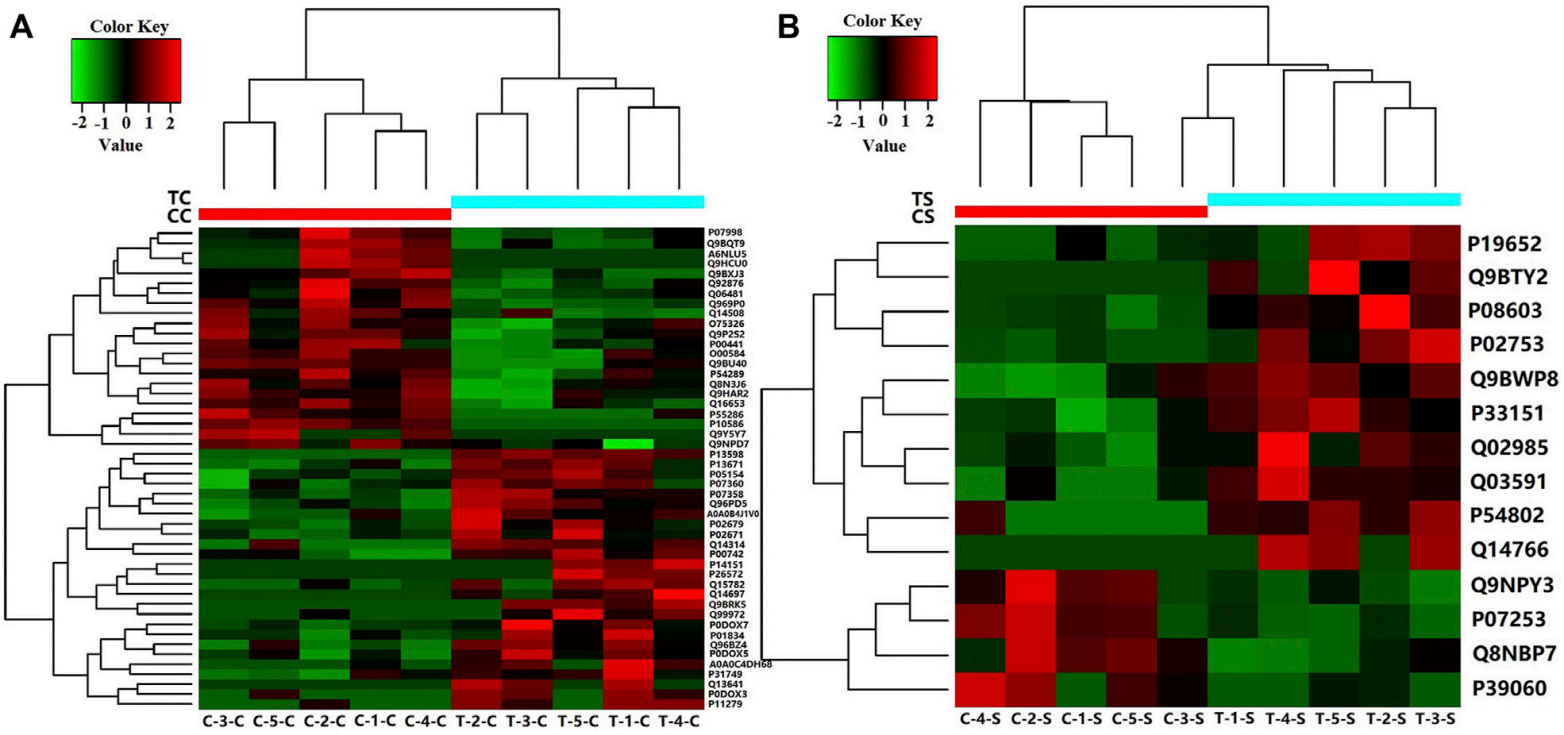
Heat map showing levels of differentially expressed proteins for which relative quantitative values were obtained for five RRMS patients and five ONIND patients (Fold Change = 1.5). **(A)** Differentially expressed proteins in RRMS CSF. **(B)** Differentially expressed proteins in RRMS serum. CC(C-1-C, C-2-C, C-3-C, C-4-C, C-5-C), CSF from ONIND; TC (T-1-C, T-2-C, T-3-C, T-4-C, T-5-C), CSF from RRMS patients; CS(C-1-S, C-2-S, C-3-S, C-4-S, C-5-S), serum from ONIND; TS(C-1-C, C-2-C, C-3-C, C-4-C, C-5-C), serum from RRMS patients. The horizontal coordinate represents the symbol of the serum and CSF of each sample.

If the relative abundance of differentially expressed proteins was defined as greater than 1.50 times or less than 0.67 times, the *p*-value < 0.05. There were 48 differential proteins in CSF samples from RRMS and ONIND groups, and 14 differential proteins in serum samples (Supplementary Table S2; Supplementary Table S4). The intensity changes of 48 differentially expressed CSF proteins are shown in Figure 1A as a heat map. The intensity changes of 14 differentially expressed serum proteins are shown in Figure 1B.

### 3.3 Bioinformatics Analysis of Differentially Expressed Proteins

#### 3.3.1 The GO Analysis

**FIGURE 2:**
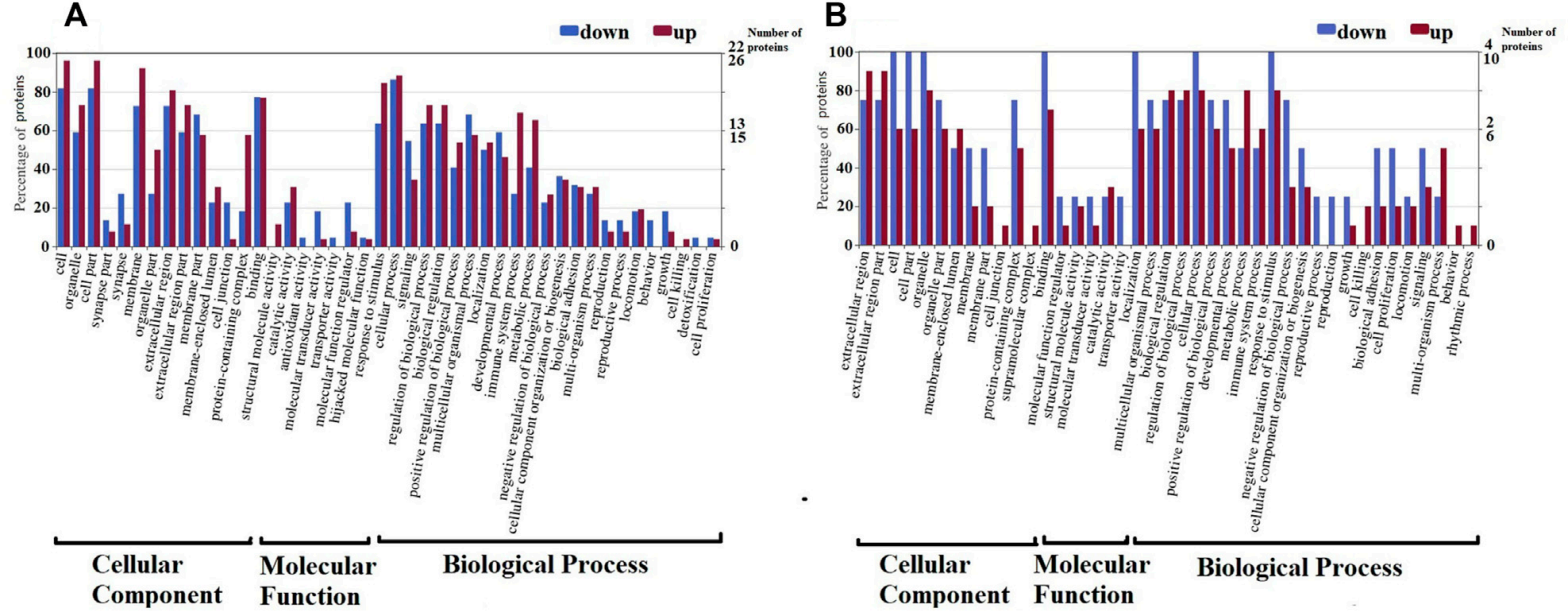
**G**ene Ontology (GO) classification of differentially expressed proteins by label-free quantitative proteomics experiments between RRMS and ONIND (Fold change = 1.5). The differentially expressed proteins are grouped into three hierarchically structured GO terms: biological process, cellular component, and molecular function in CSF **(A)** and serum **(B)**. The *y*-axis indicates the number and percent of proteins in each GO term.

The function of 48 CSF differential proteins and 14 serum differential proteins was analyzed by GO annotation, and they were divided into CC, MF and BP. CSF differentially identified proteins were classified into 28 major hierarchical GO classifications, including 2CC, 19MF, and 7BP (Figure 2). Serum identified proteins were classified into 24 major hierarchical GO classifications, including 2CC, 16MF, and 6BP (Figure 2). The CC class GO grade of CSF differential proteins is the same as that of serum. One part of CC’s function is a macromolecular complex of various proteins in cells, and the other part is constituent parts of organisms in cells. The catalytic activity, structural molecule activity, transporter activity, binding, molecular transducer activity and molecular function regulator of MF GO function of CSF differential proteins were consistent with those of serum. Antioxidant activity is a unique MF GO function of the differential proteins in CSF, and the low expression of SOD1 with antioxidant activity in CSF results in this result. The reproduction, immune, behavior, metabolic process, cellular process, biological adhesion, development process, stimulation response and regulation process of BP GO function of CSF differential proteins are consistent with that of serum. Cell growth, biological process and detoxification process are the unique BP GO functions of CSF differential proteins. The rhythmic process is the unique BP GO function of serum differential proteins.

**FIGURE 3:**
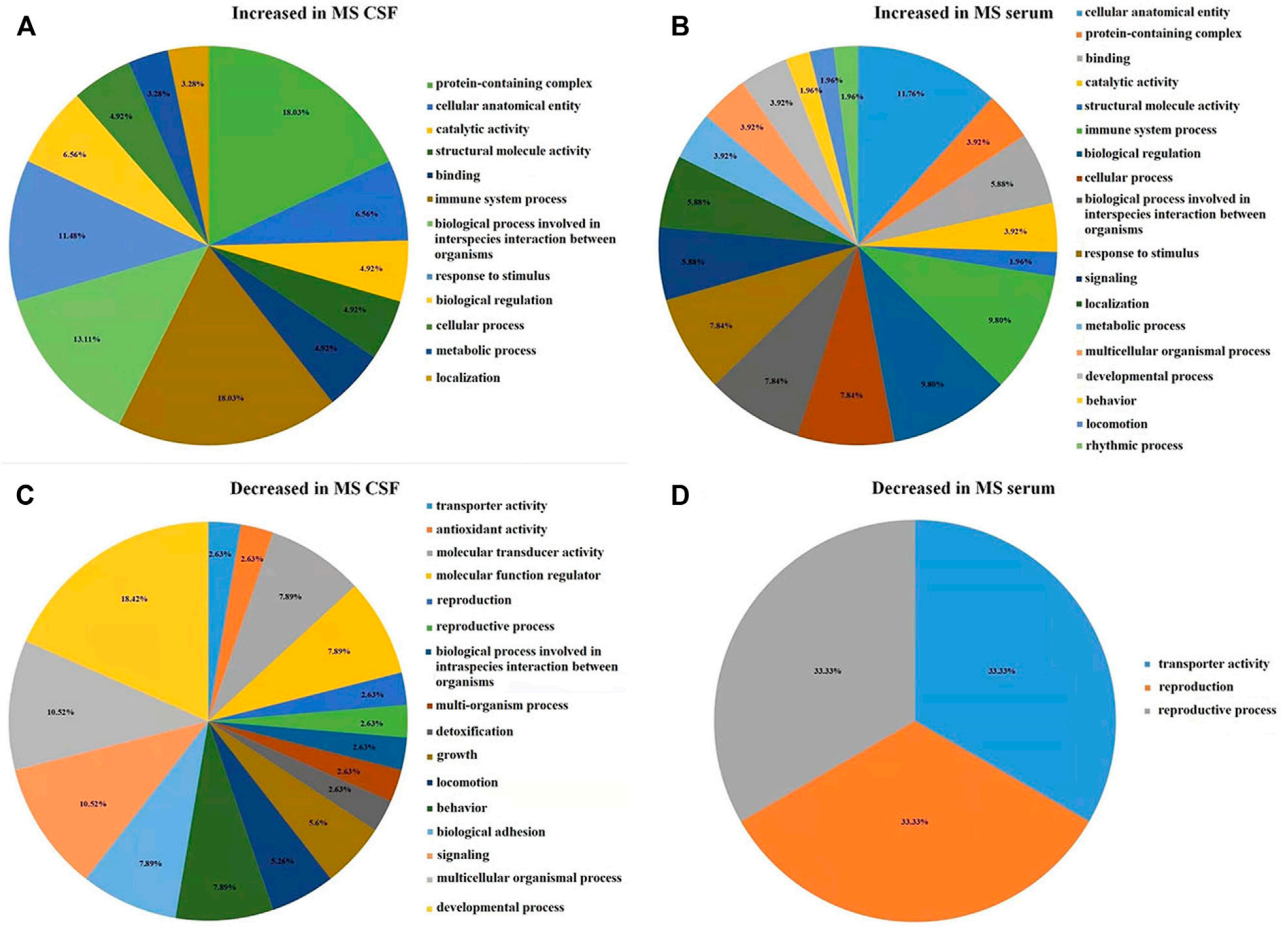
The figure is calculated by the difference between the number of highly expressed and the number of low-expressed proteins in each GO item (Fold change = 1.5). **(A)** In RRMS CSF, the number of highly expressed proteins was higher than the number of low-expressed proteins in GO entries. **(B)** In RRMS serum, the number of highly expressed proteins was higher than the number of low-expressed proteins in GO entries. **(C)** In RRMS CSF, the number of low-expressed proteins was higher than the number of highly expressed proteins in GO entries. **(D)** In RRMS serum, the number of low-expressed proteins was higher than the number of highly expressed proteins in GO entries.

In CSF of RRMS patients, GO function with more highly expressed proteins than with less expressed proteins (Figure 3A) is mainly related to immune-inflammatory responses, such as protein processing, catalytic activity, cellular processes, stimulus responses, defense against stimuli and acute inflammatory responses. The GO function in which the number of low-expressed proteins is greater than that of high-expressed proteins (Figure 3C) is mainly related to neuronal development, including nerve cell reproduction, axon development, involving chemotaxis, transport, adhesion, antioxidant and detoxification functions. This result is similar to the GO function result of serum differential proteins (Figures 3B, D). The results of GO enrichment analysis of CSF and serum differential proteins were mostly related to immune inflammatory response and nerve cell regeneration (Supplementary Table S5, Supplementary Table S6).

#### 3.3.2 The KEGG and Enrichment Analyses

**FIGURE 4:**
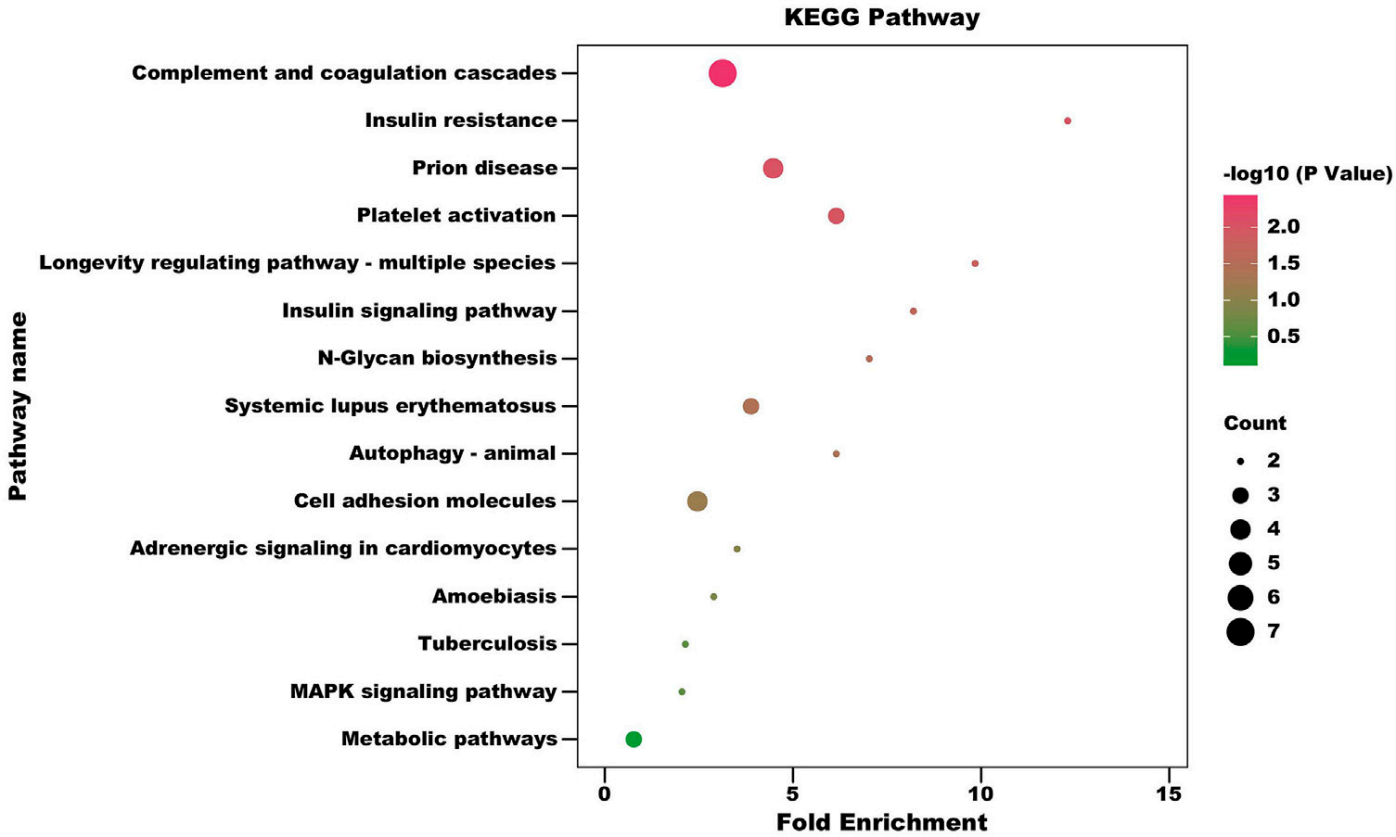
Pathway enrichment bubble diagram of differential expressed proteins of CSF (Fold change = 1.5). The horizontal axis represents fold enrichment: multiples of the total proportion of differentially expressed proteins in the KEGG signaling pathway relative to the proportion of identified proteins in this signaling pathway; The ordinate represents KEGG term description; Bubble size indicates the number of differential proteins in the KEGG signaling pathway. Fisher exact test *p* value: *p* values of the enrichment test obtained using Fisher’s exact test.

In order to obtain more information about the biological functions of differential proteins, KEGG enrichment analysis was performed on the differential proteins identified in CSF and serum, respectively. Hypergeometric tests were used to identify pathways that are significantly enriched in differential proteins compared to all identified protein backgrounds to identify the most important biochemical metabolic pathways and signal transduction pathways involved in differential proteins in CSF and serum of RRMS patients. Using a standard of *p* < 0.05 and impact factor threshold >0, KEGG enrichment of differential proteins in CSF is shown in Figure 4; Supplementary Table S7. Analysis showed that the complement and coagulation cascades (*p* = 0.0036), insulin resistance (*p* = 0.00898), prion disease (*p* = 0.00909), platelet activation (*p* = 0.01002), longevity regulating pathway - multiple species (*p* = 0.01460), insulin signaling pathway (*p* = 0.02137), N-Glycan biosynthesis (*p* = 0.02919), systemic lupus erythematosus (*p* = 0.03672) and autophagy - animal (*p* = 0.03798) were significantly associated with the occurrence of RRMS.

### 3.4 Genome-wide Exon Analysis

A total of 2,759 exon variants were detected in eight RRMS patients. Among them, nine genes were identical to the CSF differential protein: CFH, C8B, NRCAM, VTN, C7, NID1, KLKB1, C8G, GPX3 (Table 3). Similarly, there were nine genes identical with the serum differential protein: CFH, LTBP1, FN1, CD93, AHSG, PCSK9, C8G, COL18A1, CFI (Table 3).

**TABLE 3:** Genetic mutations identical to the CSF and serum differential protein. (Fold change = 1.2).

	GeneID	Symbol	Description
CSF	ENSG00000000971.15	CFH	complement factor H
	ENSG00000021852.12	C8B	complement C8 beta chain
	ENSG00000091129.19	NRCAM	neuronal cell adhesion molecule
	ENSG00000109072.13	VTN	vitronectin
	ENSG00000112936.18	C7	complement C7
	ENSG00000116962.14	NID1	nidogen 1
	ENSG00000164344.15	KLKB1	kallikrein B1
	ENSG00000176919.11	C8G	complement C8 gamma chain
	ENSG00000211445.11	GPX3	glutathione peroxidase 3
serum	ENSG00000000971.15	CFH	complement factor H
	ENSG00000049323.15	LTBP1	latent transforming growth factor beta binding protein 1
	ENSG00000115414.18	FN1	fibronectin 1
	ENSG00000125810.9	CD93	CD93 molecule
	ENSG00000145192.12	AHSG	alpha 2-HS glycoprotein
	ENSG00000169174.10	PCSK9	proprotein convertase subtilisin/kexin type 9
	ENSG00000176919.11	C8G	complement C8 gamma chain
	ENSG00000182871.14	COL18A1	collagen type XVIII alpha 1 chain
	ENSG00000205403.12	CFI	complement factor I

## 4 Discussion

To identify the potential biomarkers in CSF and serum of patients with RRMS, we performed differential analysis of CSF and serum proteomics in the control group and RRMS patients and obtained 73 CSF significantly differential proteins and 22 serum significantly different proteins. However, in our study, MMP2, C8G (Kroksveen et al., 2017) and CFH (Liu et al., 2009) were found to be highly expressed in both CSF and serum, which indicated that the abnormal expression of these proteins was highly correlated with the occurrence of RRMS. MMP2, a protein that reflects high levels of grey matter damage in MS patients (Magliozzi et al., 2018), has been confirmed to cause blood-brain barrier disruption, basal layer remodeling (Aksoy et al., 2016). It is well known that the occurrence of MS is associated with inflammatory demyelination of the central nervous system. Neuroinflammation is considered to be the triggering mechanism of axon regeneration in the mammalian central nervous system, and MMP2 is an important factor connecting inflammation and axon regeneration (Andries et al., 2021). CFH is an important member of the regulator of complement activation (RCA) group of proteins encoded within the RCA gene locus on chromosome 21 (chr 1q21–1q32) (Lukiw and Alexandrov, 2012). CFH normally acts as a critical complement and innate immune system repressor, as a specific inhibitor of the C3 to C3b transition in the complement pathway (Finehout et al., 2005; Abdel Rasol et al., 2015). Human C8G gene is located on chromosome 9q34.3, which harbours a cluster of lipocalin genes that encode a protein family that interacts with small bioactive molecules and specific cell-surface receptors (Lögdberg and Wester, 2000). C8G plays an anti-inflammatory role by inhibiting the pro-inflammatory sphingosine 1-phosphate receptor two signaling pathway in microglia (Kim et al., 2021).

In order to verify the correlation between some of the above-mentioned differential proteins and MS, genome-wide exon sequencing was performed on eight additional patients with RRMS. Nine of the 2,759 mutated genes were consistent with CSF differential proteins: CFH, C8B, NRCAM, VTN, C7, NID1, KLKB1, C8G, GPX3. Nine genes were consistent with serum differential proteins: CFH, LTBP1, FN1, CD93, AHSG, PCSK9, C8G, COL18A1, CFI. Furthermore, the differential expression of these 16 proteins was closely related to the occurrence of RRMS. However, it is still unknown whether the differential expression of proteins is caused by these gene mutations (Wang W. et al., 2022) (Peng et al., 2021b) (Wang et al., 2021) (Wang T. et al., 2022). In the future, we will link gene mutations with differential expression of proteins to study the pathogenesis of diseases.

Previous studies were conducted on CSF and blood proteomics of MS patients, and the research of them were proved to be consistent with our study results. Firstly, FAM3C (Hinsinger et al., 2015) (Kroksveen et al., 2017) (Opsahl et al., 2016), CHI3L2 (Hinsinger et al., 2015) (Kroksveen et al., 2017) (Opsahl et al., 2016) (Guldbrandsen et al., 2020) (Jankovska et al., 2020), NRXN2 (Kroksveen et al., 2017) (Opsahl et al., 2016) (Guldbrandsen et al., 2020), RNASET2 (Kroksveen et al., 2017) (Opsahl et al., 2016) (Guldbrandsen et al., 2020), APLP1 (Kroksveen et al., 2017) (Liu et al., 2009) (Opsahl et al., 2016) (Guldbrandsen et al., 2020), CST3 (Kroksveen et al., 2017) (Liu et al., 2009) (Jankovska et al., 2020) (Füvesi et al., 2012), KLK6 (Kroksveen et al., 2017) (Liu et al., 2009) (Guldbrandsen et al., 2020) (Kroksveen et al., 2013), and IGKC (Hinsinger et al., 2015) (Kroksveen et al., 2017) (Liu et al., 2009) (Jankovska et al., 2020) have been identified as biomarkers for MS, which are consistent with the results of CSF in our study. In addition, the confirmed significant proteins C3 (Malekzadeh et al., 2019) (Wallin et al., 2015), CLU (Liu et al., 2009) (Kroksveen et al., 2013), LYZ (Kroksveen et al., 2017) (Jankovska et al., 2020), CFI (Jankovska et al., 2020) (Wallin et al., 2015) and FN1 (Wallin et al., 2015) have been also proved to be blood biomarkers. What’s more, three previous studies using the experimental autoimmune encephalomyelitis (EAE) mouse model have identified several protein markers identical to our MS study. For example, differentially expressed protein C3 was found in spinal cord tissue, brain tissue (Hasan et al., 2019), CSF (Rosenling et al., 2012) and peripheral blood (Dagley et al., 2014) of EAE mice. In addition, significant protein FGA and FGG were confirmed in spinal cord, brain tissue (Hasan et al., 2019) and CSF (Rosenling et al., 2012). FN1 was identified in spinal cord, brain tissue (Hasan et al., 2019) and peripheral blood (Dagley et al., 2014). AHSG was identified in peripheral blood (Dagley et al., 2014) and CSF (Rosenling et al., 2012).

GO analysis was performed on the significantly different proteins in serum and CSF to clarify their roles, and the results are as follows: the occurrence of MS was mainly related to immune inflammatory response and neural cell development disorder (Kroksveen et al., 2017). The occurrence of immune responses includes protein processing, catalytic activity, cellular processes, stimulating response, stimulating defense, and acute inflammatory response. Nerve cell growth and development include nerve cell reproduction, axon development, involving chemotaxis, transport, adhesion, antioxidant and detoxification functions.

In the previous multipath analysis of different MS-GWAS datasets, 10 shared genetic pathways were identified by validation of disordered genes from seven human MS case-control expression datasets (Liu et al., 2017). In the current study, KEGG analysis of differentially expressed proteins in serum and CSF was performed to obtain several genetic pathways related to MS, and the previous four genetic pathways were verified to be related to MS: Toxoplasmosis, chemokine signaling pathway, focal adhesion, acute myeloid leukemia, JAK-STAT signaling pathway, protein processing in endoplasmic reticulum.

A comprehensive and unbiased study of RRMS was conducted, identifying a range of CSF and serum proteins associated with RRMS. This study confirmed many proteins previously observed to be involved in RRMS, and also revealed three proteins that play an important role in the development of RRMS: MMP2, C8G, and CFH. These results are valuable for the design of targeted proteomic analyses of RRMS for external validation, and further studies are needed to verify the clinical efficacy of significant proteins. Pathways and cluster analysis of meaningful protein involvement in CSF and serum provide insights into the biological processes that engender RRMS and improve our understanding of the underlying mechanisms underlying the development of RRMS. The sample size of the experimental group and the control group in this study is small, but it provides a good preliminary basis for further research. Our further study plan is will conducted in larger sample size population and compare the diagnostic effects of combined biomarkers. In addition, we will also study biomarkers related to the severity and course of MS disease, so as to provide some guidance for the clinical treatment of this disease.

## 5 Conclusion

In summary, the study demonstrated 73 proteins in CSF and 22 proteins in serum were significantly differentially expressed in the RRMS set compared with the controls. What is more, MMP2, C8G and CFH may participate in the pathogenesis of RRMS, which are the potential diagnostic biomarkers of the disease.

## Data Availability

The mass spectrometry proteomics data have been deposited to the ProteomeXchange Consortium (http://proteomecentral.proteomexchange.org) *via* the iProX partner repository with the dataset identifier PXD032287. The raw sequence data of gene have been deposited in the Genome Sequence Archive in National Genomics Data Center, China National Center for Bioinformation / Beijing Institute of Genomics, Chinese Academy of Sciences (GSA-Human: HRA002221) that are publicly accessible at https://ngdc.cncb.ac.cn/gsa-human.
