# Supplementary material for: Label-free Quantitative Proteomic Analysis of Cerebrospinal Fluid and Serum in Patients With Relapse-Remitting Multiple Sclerosis
Source: Front Genet. 2022 Apr 27;13:892491. doi: 10.3389/fgene.2022.892491 (PMC9092947; doi:10.3389/fgene.2022.892491)
Supplement: Supplementary file 1 [file DataSheet1.docx]

**Supplementary Table S1** Seventy-three differentially expressed proteins in CSF.（Fold change=1.2）.

| Gene | Protein name | Uniprot | Ratio | *p*-value |
| --- | --- | --- | --- | --- |
| GANAB | Neutral alpha-glucosidase AB | Q14697 | 6.4646 | 0.0363 |
| ICAM2 | Intercellular adhesion molecule 2 | P13598 | 5.7544 | 1.25E-07 |
| SDF4 | 45 kDa calcium-binding protein | Q9BRK5 | 5.5834 | 0.0064 |
| IGKV2-24 | Immunoglobulin kappa variable 2-24 | A0A0C4DH68 | 4.937 | 0.03078 |
| CHI3L2 | Chitinase-3-like protein 2 | Q15782 | 4.8481 | 0.0194 |
| MYOC | Myocilin | Q99972 | 4.6427 | 0.0466 |
| TPBG | Trophoblast glycoprotein | Q13641 | 4.1395 | 0.0454 |
| SELL | L-selectin | P14151 | 4.0138 | 0.0471 |
| MGAT1 | Alpha-1,3-mannosyl-glycoprotein 2-beta-N-acetylglucosaminyltransferase | P26572 | 3.8637 | 0.0487 |
| PLD4 | 5'-3' exonuclease PLD4 | Q96BZ4 | 3.7912 | 0.0070 |
| FGL2 | Fibroleukin | Q14314 | 3.3094 | 0.0085 |
| LAMP1 | Lysosome-associated membrane glycoprotein 1 | P11279 | 3.3020 | 0.0282 |
|  | Immunoglobulin delta heavy chain | P0DOX3 | 3.0456 | 0.0358 |
| F10 | Coagulation factor X | P00742 | 2.8451 | 0.0070 |
| IGHV3-15 | Immunoglobulin heavy variable 3-15 | A0A0B4J1V0 | 2.6637 | 0.0137 |
| FGG | Fibrinogen gamma chain | P02679 | 2.5037 | 0.0230 |
|  | Immunoglobulin kappa light chain | P0DOX7 | 2.5006 | 0.0288 |
| SERPINA5 | Plasma serine protease inhibitor | P05154 | 2.1029 | 0.0071 |
| C8B | Complement component C8 beta chain | P07358 | 2.0619 | 0.0036 |
| FGA | Fibrinogen alpha chain | P02671 | 2.0618 | 0.0497 |
| AKT1 | RAC-alpha serine/threonine-protein kinase | P31749 | 1.7696 | 0.0494 |
|  | Immunoglobulin gamma-1 heavy chain | P0DOX5 | 1.7015 | 0.0245 |
| PGLYRP2 | N-acetylmuramoyl-L-alanine amidase | Q96PD5 | 1.6209 | 0.0039 |
| IGKC | Immunoglobulin kappa constant | P01834 | 1.6046 | 0.0452 |
| C8G | Complement component C8 gamma chain | P07360 | 1.5666 | 0.0275 |
| C6 | Complement component C6 | P13671 | 1.5612 | 0.0063 |
| C2 | Complement C2 | P06681 | 1.4902 | 0.0494 |
| KLKB1 | Plasma kallikrein | P03952 | 1.4416 | 0.0385 |
| C7 | Complement component C7 | P10643 | 1.4107 | 0.0228 |
| VTN | Vitronectin | P04004 | 1.3820 | 0.0429 |
| AFM | Afamin | P43652 | 1.3457 | 0.0451 |
| MMP2 | 72 kDa type IV collagenase | P08253 | 1.3060 | 0.0153 |
| ATRN | Attractin | O75882 | 1.2609 | 0.0311 |
| F2 | Prothrombin | P00734 | 1.2443 | 0.0213 |
| CFH | Complement factor H | P08603 | 1.1395 | 0.0291 |
| FAM3C | Protein FAM3C | Q92520 | 0.8178 | 0.0433 |
| CLSTN1 | Calsyntenin-1 | O94985 | 0.8155 | 0.0428 |
| GPX3 | Glutathione peroxidase 3 | P22352 | 0.8031 | 0.0448 |
| FBLN5 | Fibulin-5 | Q9UBX5 | 0.7955 | 0.0472 |
| ECM1 | Extracellular matrix protein 1 | Q16610 | 0.7759 | 0.0361 |
| NRCAM | Neuronal cell adhesion molecule | Q92823 | 0.7759 | 0.0495 |
| NUCB1 | Nucleobindin-1 | Q02818 | 0.7736 | 0.0109 |
| NELL2 | Protein kinase C-binding protein NELL2 | Q99435 | 0.7224 | 0.0360 |
| NID1 | Nidogen-1 | P14543 | 0.7140 | 0.0421 |
| APLP1 | Amyloid-like protein 1 | P51693 | 0.7084 | 0.0185 |
| NEGR1 | Neuronal growth regulator 1 | Q7Z3B1 | 0.7077 | 0.0292 |
| CRTAC1 | Cartilage acidic protein 1 | Q9NQ79 | 0.7030 | 0.0193 |
| CANT1 | Soluble calcium-activated nucleotidase 1 | Q8WVQ1 | 0.7016 | 0.0125 |
| CST3 | Cystatin-C | P01034 | 0.6982 | 0.0251 |
| APP | Amyloid-beta precursor protein | P05067 | 0.6881 | 0.0420 |
| CADM1 | Cell adhesion molecule 1 | Q9BY67 | 0.6732 | 0.0120 |
| CACNA2D1 | Voltage-dependent calcium channel subunit alpha-2/delta-1 | P54289 | 0.6566 | 0.0315 |
| RNASET2 | Ribonuclease T2 | O00584 | 0.6423 | 0.0144 |
| CADM2 | Cell adhesion molecule 2 | Q8N3J6 | 0.6385 | 0.0285 |
| RNASE1 | Ribonuclease pancreatic | P07998 | 0.6141 | 0.0265 |
| SEMA7A | Semaphorin-7A | O75326 | 0.6129 | 0.0451 |
| NRN1 | Neuritin | Q9NPD7 | 0.6097 | 0.0350 |
| IGSF8 | Immunoglobulin superfamily member 8 | Q969P0 | 0.6034 | 0.0013 |
| KLK6 | Kallikrein-6 | Q92876 | 0.5907 | 0.0190 |
| NRXN2 | Neurexin-2 | Q9P2S2 | 0.5779 | 0.0158 |
| SOD1 | Superoxide dismutase [Cu-Zn] | P00441 | 0.5647 | 0.0186 |
| MOG | Myelin-oligodendrocyte glycoprotein | Q16653 | 0.5622 | 0.0033 |
| APLP2 | Amyloid-like protein 2 | Q06481 | 0.5310 | 0.0465 |
| ADGRL3 | Adhesion G protein-coupled receptor L3 | Q9HAR2 | 0.4921 | 0.0222 |
| CHRDL1 | Chordin-like protein 1 | Q9BU40 | 0.4039 | 0.0063 |
| CLSTN3 | Calsyntenin-3 | Q9BQT9 | 0.3377 | 0.0442 |
| WFDC2 | WAP four-disulfide core domain protein 2 | Q14508 | 0.2993 | 0.0264 |
| CDH8 | Cadherin-8 | P55286 | 0.2600 | 0.0046 |
| VSTM2B | V-set and transmembrane domain-containing protein 2B | A6NLU5 | 0.2542 | 0.0473 |
| CD248 | Endosialin | Q9HCU0 | 0.2471 | 0.0453 |
| LYVE1 | Lymphatic vessel endothelial hyaluronic acid receptor 1 | Q9Y5Y7 | 0.2391 | 0.0461 |
| C1QTNF4 | Complement C1q tumor necrosis factor-related protein 4 | Q9BXJ3 | 0.1718 | 0.0037 |
| PTPRF  CFH, FC=1.1395, P=0.0291. Although CFH does not meet the standard of FC≥1.2, this protein is of great significance to the occurrence of MS, so we still include it in this table. | Receptor-type tyrosine-protein phosphatase F | P10586 | 0.1577 | 2.40E-06 |

**Supplementary Table S2** Forty-eight differentially expressed proteins in CSF.（Fold change=1.5）

| Gene | Protein name | Uniprot | Ratio | *p*-value |
| --- | --- | --- | --- | --- |
| GANAB | Neutral alpha-glucosidase AB | Q14697 | 6.4646 | 0.0363 |
| ICAM2 | Intercellular adhesion molecule 2 | P13598 | 5.7544 | 1.25E-07 |
| SDF4 | 45 kDa calcium-binding protein | Q9BRK5 | 5.5834 | 0.0064 |
| IGKV2-24 | Immunoglobulin kappa variable 2-24 | A0A0C4DH68 | 4.9370 | 0.0307 |
| CHI3L2 | Chitinase-3-like protein 2 | Q15782 | 4.8481 | 0.0194 |
| MYOC | Myocilin | Q99972 | 4.6427 | 0.0466 |
| TPBG | Trophoblast glycoprotein | Q13641 | 4.1395 | 0.0454 |
| SELL | L-selectin | P14151 | 4.0138 | 0.0471 |
| MGAT1 | Alpha-1,3-mannosyl-glycoprotein 2-beta-N-acetylglucosaminyltransferase | P26572 | 3.8637 | 0.0487 |
| PLD4 | 5'-3' exonuclease PLD4 | Q96BZ4 | 3.7912 | 0.0070 |
| FGL2 | Fibroleukin | Q14314 | 3.3094 | 0.0085 |
| LAMP1 | Lysosome-associated membrane glycoprotein 1 | P11279 | 3.3020 | 0.0282 |
|  | Immunoglobulin delta heavy chain | P0DOX3 | 3.0456 | 0.0358 |
| F10 | Coagulation factor X | P00742 | 2.8451 | 0.0070 |
| IGHV3-15 | Immunoglobulin heavy variable 3-15 | A0A0B4J1V0 | 2.6637 | 0.0139 |
| FGG | Fibrinogen gamma chain | P02679 | 2.5037 | 0.0230 |
|  | Immunoglobulin kappa light chain | P0DOX7 | 2.5006 | 0.0288 |
| SERPINA5 | Plasma serine protease inhibitor | P05154 | 2.1029 | 0.0071 |
| C8B | Complement component C8 beta chain | P07358 | 2.0619 | 0.0036 |
| FGA | Fibrinogen alpha chain | P02671 | 2.0618 | 0.0497 |
| AKT1 | RAC-alpha serine/threonine-protein kinase | P31749 | 1.7696 | 0.0494 |
|  | Immunoglobulin gamma-1 heavy chain | P0DOX5 | 1.7015 | 0.0245 |
| PGLYRP2 | N-acetylmuramoyl-L-alanine amidase | Q96PD5 | 1.6209 | 0.0039 |
| IGKC | Immunoglobulin kappa constant | P01834 | 1.6046 | 0.0452 |
| C8G | Complement component C8 gamma chain | P07360 | 1.5666 | 0.0275 |
| C6 | Complement component C6 | P13671 | 1.5612 | 0.0063 |
| CACNA2D1 | Voltage-dependent calcium channel subunit alpha-2/delta-1 | P54289 | 0.6566 | 0.0315 |
| RNASET2 | Ribonuclease T2 | O00584 | 0.6423 | 0.0144 |
| CADM2 | Cell adhesion molecule 2 | Q8N3J6 | 0.6385 | 0.0285 |
| RNASE1 | Ribonuclease pancreatic | P07998 | 0.6141 | 0.0265 |
| SEMA7A | Semaphorin-7A | O75326 | 0.6129 | 0.0451 |
| NRN1 | Neuritin | Q9NPD7 | 0.6097 | 0.0350 |
| IGSF8 | Immunoglobulin superfamily member 8 | Q969P0 | 0.6034 | 0.0013 |
| KLK6 | Kallikrein-6 | Q92876 | 0.5907 | 0.0190 |
| NRXN2 | Neurexin-2 | Q9P2S2 | 0.5779 | 0.0158 |
| SOD1 | Superoxide dismutase [Cu-Zn] | P00441 | 0.5647 | 0.0186 |
| MOG | Myelin-oligodendrocyte glycoprotein | Q16653 | 0.5622 | 0.0033 |
| APLP2 | Amyloid-like protein 2 | Q06481 | 0.5310 | 0.0465 |
| ADGRL3 | Adhesion G protein-coupled receptor L3 | Q9HAR2 | 0.4921 | 0.0222 |
| CHRDL1 | Chordin-like protein 1 | Q9BU40 | 0.4039 | 0.0063 |
| CLSTN3 | Calsyntenin-3 | Q9BQT9 | 0.3377 | 0.0442 |
| WFDC2 | WAP four-disulfide core domain protein 2 | Q14508 | 0.2993 | 0.0264 |
| CDH8 | Cadherin-8 | P55286 | 0.2600 | 0.0046 |
| VSTM2B | V-set and transmembrane domain-containing protein 2B | A6NLU5 | 0.2542 | 0.0473 |
| CD248 | Endosialin | Q9HCU0 | 0.2471 | 0.0453 |
| LYVE1 | Lymphatic vessel endothelial hyaluronic acid receptor 1 | Q9Y5Y7 | 0.2391 | 0.0461 |
| C1QTNF4 | Complement C1q tumor necrosis factor-related protein 4 | Q9BXJ3 | 0.1718 | 0.0037 |
| PTPRF | Receptor-type tyrosine-protein phosphatase F | P10586 | 0.1577 | 2.40E-06 |

**Supplementary Table S3** Twenty-two differentially expressed proteins in serum.（Fold change=1.2）

| Gene | Protein name | Uniprot | Ratio | *p*-value |
| --- | --- | --- | --- | --- |
| FN1 | Fibronectin | P02751 | 11.0432 | 0.0119 |
| FUCA2 | Plasma alpha-L-fucosidase | Q9BTY2 | 9.0848 | 0.0362 |
| LTBP1 | Latent-transforming growth factor beta-binding protein 1 | Q14766 | 3.6028 | 0.0409 |
| CFHR1 | Complement factor H-related protein 1 | Q03591 | 3.2821 | 0.0072 |
| NAGLU | Alpha-N-acetylglucosaminidase | P54802 | 3.2012 | 0.0044 |
| CFH | Complement factor H | P08603 | 3.0811 | 0.0126 |
| CFHR3 | Complement factor H-related protein 3 | Q02985 | 2.6289 | 0.0410 |
| ORM2 | Alpha-1-acid glycoprotein 2 | P19652 | 1.9744 | 0.0375 |
| CDH5 | Cadherin-5 | P33151 | 1.7851 | 0.0044 |
| COLEC11 | Collectin-11 | Q9BWP8 | 1.5136 | 0.0074 |
| MMP2 | 72 kDa type IV collagenase | P08253 | 1.4986 | 0.0340 |
| LYZ | Lysozyme C | P61626 | 1.4337 | 0.0463 |
| C8G | Complement component C8 gamma chain | P07360 | 1.3264 | 0.0238 |
| CLU | Clusterin | P10909 | 1.2789 | 0.0477 |
| C3 | Complement C3 | P01024 | 0.7908 | 0.0485 |
| CFI | Complement factor I | P05156 | 0.7653 | 0.0264 |
| FETUB | Fetuin-B | Q9UGM5 | 0.6928 | 0.0339 |
| AHSG | Alpha-2-HS-glycoprotein | P02765 | 0.6819 | 0.0115 |
| RBP4 | Retinol-binding protein 4 | P02753 | 0.6519 | 0.0140 |
| PCSK9 | Proprotein convertase subtilisin/kexin type 9 | Q8NBP7 | 0.5701 | 0.0148 |
| CD93 | Complement component C1q receptor | Q9NPY3 | 0.5246 | 0.0220 |
| COL18A1 | Collagen alpha-1(XVIII) chain | P39060 | 0.2247 | 0.0386 |

**Supplementary Table S4** Fourteen differentially expressed proteins in serum.（Fold change=1.5）

| Gene | Protein name | Uniprot | Ratio | *p*-value |
| --- | --- | --- | --- | --- |
| FN1 | Fibronectin | P02751 | 11.0432 | 0.0119 |
| FUCA2 | Plasma alpha-L-fucosidase | Q9BTY2 | 9.0848 | 0.0362 |
| LTBP1 | Latent-transforming growth factor beta-binding protein 1 | Q14766 | 3.6028 | 0.0409 |
| CFHR1 | Complement factor H-related protein 1 | Q03591 | 3.2821 | 0.0072 |
| NAGLU | Alpha-N-acetylglucosaminidase | P54802 | 3.2012 | 0.0044 |
| CFH | Complement factor H | P08603 | 3.0811 | 0.0126 |
| CFHR3 | Complement factor H-related protein 3 | Q02985 | 2.6289 | 0.0410 |
| ORM2 | Alpha-1-acid glycoprotein 2 | P19652 | 1.9744 | 0.0375 |
| CDH5 | Cadherin-5 | P33151 | 1.7851 | 0.0044 |
| COLEC11 | Collectin-11 | Q9BWP8 | 1.5136 | 0.0074 |
| RBP4 | Retinol-binding protein 4 | P02753 | 0.6519 | 0.0140 |
| PCSK9 | Proprotein convertase subtilisin/kexin type 9 | Q8NBP7 | 0.5701 | 0.0148 |
| CD93 | Complement component C1q receptor | Q9NPY3 | 0.5246 | 0.0220 |
| COL18A1 | Collagen alpha-1(XVIII) chain | P39060 | 0.2247 | 0.0386 |

**Supplementary Table S5**  GO enrichment analysis of the CSF identified proteins.（Fold change=1.5）

| GO_ID | Category | *p*-value | Corrected *p*-value | No. | Gene |
| --- | --- | --- | --- | --- | --- |
| GO:0045907 | positive regulation of vasoconstriction | 0.001 | 0.230 | 3 | P02679, P02671, P31749 |
| GO:0019229 | regulation of vasoconstriction | 0.002 | 0.230 | 3 | P31749, P02679, P02671 |
| GO:0005886 | plasma membrane | 0.002 | 0.230 | 34 | A0A0B4J1V0, P14151, P0DOX7, P13598, P0DOX5, Q06481, P02671, P11279, P02679, P05154, P00441, P07360, Q969P0, Q9P2S2, Q13641, Q9HAR2, Q9BRK5, P10586, Q9NPD7, A0A0C4DH68, P00742, P01834, Q9BQT9, P54289, Q9HCU0, Q16653, Q8N3J6, P31749, O75326, P55286, P0DOX3, Q9Y5Y7, P07358, P13671 |
| GO:0032287 | peripheral nervous system myelin maintenance | 0.002 | 0.230 | 2 | P31749, P00441 |
| GO:1903409 | reactive oxygen species biosynthetic process | 0.002 | 0.230 | 2 | P31749, P00441 |
| GO:0016020 | membrane | 0.003 | 0.230 | 40 | P31749, P26572, Q96BZ4, Q8N3J6, Q16653, Q9HCU0, Q9BQT9, P54289, P07358, P13671, Q9Y5Y7, P0DOX3, P55286, O75326, Q14697, Q969P0, P07360, P00441, P02679, P05154, P11279, P02671, P0DOX5, Q06481, P13598, P14151, P0DOX7, Q96PD5, A0A0B4J1V0, Q99972, A6NLU5, P01834, P00742, A0A0C4DH68, Q9NPD7, P10586, Q9BRK5, Q9P2S2, Q13641, Q9HAR2 |
| GO:0071944 | cell periphery | 0.003 | 0.230 | 34 | P00742, P01834, A0A0C4DH68, Q9NPD7, P10586, Q9BRK5, Q13641, Q9P2S2, Q9HAR2, P07360, Q969P0, P00441, P05154, P02679, P02671, P11279, P0DOX5, Q06481, P13598, P14151, P0DOX7, A0A0B4J1V0, P07358, P13671, Q9Y5Y7, P0DOX3, P55286, O75326, P31749, Q8N3J6, Q16653, Q9HCU0, P54289, Q9BQT9 |
| GO:0022011 | myelination in peripheral nervous system | 0.003 | 0.230 | 3 | P31749, P00441, Q99972 |
| GO:0032292 | peripheral nervous system axon ensheathment | 0.003 | 0.230 | 3 | P00441, P31749, Q99972 |
| GO:0005577 | fibrinogen complex | 0.003 | 0.230 | 3 | P02671, Q14314, P02679 |
| GO:0005579 | membrane attack complex | 0.003 | 0.230 | 3 | P07360, P07358, P13671 |
| GO:0046930 | pore complex | 0.003 | 0.230 | 3 | P13671, P07358, P07360 |
| GO:0009897 | external side of plasma membrane | 0.004 | 0.230 | 11 | O75326, Q16653, P14151, Q9HCU0, A0A0B4J1V0, P11279, P02671, P00742, P02679, P01834, P05154 |
| GO:0035150 | regulation of tube size | 0.005 | 0.230 | 4 | P02671, P02679, P31749, P00441 |
| GO:0035296 | regulation of tube diameter | 0.005 | 0.230 | 4 | P02671, P02679, P31749, P00441 |
| GO:0097746 | blood vessel diameter maintenance | 0.005 | 0.230 | 4 | P02671, P02679, P31749, P00441 |
| GO:0014044 | Schwann cell development | 0.005 | 0.230 | 3 | P31749, P00441, Q99972 |
| GO:0051881 | regulation of mitochondrial membrane potential | 0.005 | 0.230 | 3 | P31749, P00441, Q99972 |
| GO:1900026 | positive regulation of substrate adhesion-dependent cell spreading | 0.005 | 0.230 | 3 | Q99972, P02671, P02679 |
| GO:2000351 | regulation of endothelial cell apoptotic process | 0.005 | 0.230 | 3 | P02671, P02679, Q9HCU0 |
| GO:0035249 | synaptic transmission, glutamatergic | 0.007 | 0.250 | 2 | P55286, Q9BQT9 |
| GO:0090502 | RNA phosphodiester bond hydrolysis, endonucleolytic | 0.007 | 0.250 | 2 | O00584, P07998 |
| GO:0004521 | endoribonuclease activity | 0.007 | 0.250 | 2 | O00584, P07998 |
| GO:0016892 | endoribonuclease activity, producing 3'-phosphomonoesters | 0.007 | 0.250 | 2 | O00584, P07998 |
| GO:0045921 | positive regulation of exocytosis | 0.008 | 0.266 | 3 | P11279, P02671, P02679 |
| GO:0007272 | ensheathment of neurons | 0.010 | 0.297 | 4 | P00441, P31749, Q92876, Q99972 |
| GO:0008366 | axon ensheathment | 0.010 | 0.297 | 4 | Q99972, Q92876, P31749, P00441 |
| GO:0042552 | myelination | 0.010 | 0.297 | 4 | P31749, P00441, Q92876, Q99972 |
| GO:0014037 | Schwann cell differentiation | 0.011 | 0.298 | 3 | Q99972, P31749, P00441 |
| GO:0004518 | nuclease activity | 0.011 | 0.298 | 3 | Q96BZ4, O00584, P07998 |
| GO:0098552 | side of membrane | 0.012 | 0.298 | 11 | Q16653, P14151, O75326, Q9HCU0, A0A0B4J1V0, P02679, P00742, P05154, P01834, P02671, P11279 |
| GO:0007276 | gamete generation | 0.012 | 0.298 | 4 | P05154, Q14508, P31749, P00441 |
| GO:0016021 | integral component of membrane | 0.012 | 0.298 | 23 | A6NLU5, P07358, P13671, Q9Y5Y7, P55286, P10586, O75326, Q9P2S2, Q13641, Q9HAR2, P26572, Q96BZ4, P07360, Q969P0, Q8N3J6, Q06481, P13598, P11279, Q9HCU0, P14151, Q16653, P54289, Q9BQT9 |
| GO:0019953 | sexual reproduction | 0.013 | 0.298 | 5 | P31749, Q969P0, P00441, P05154, Q14508 |
| GO:0043217 | myelin maintenance | 0.013 | 0.298 | 2 | P31749, P00441 |
| GO:0016894 | endonuclease activity, active with either ribo- or deoxyribonucleic acids and producing 3'-phosphomonoesters | 0.013 | 0.298 | 2 | P07998, O00584 |
| GO:0045619 | regulation of lymphocyte differentiation | 0.014 | 0.298 | 3 | Q14314, P00441, Q96PD5 |
| GO:0090305 | nucleic acid phosphodiester bond hydrolysis | 0.014 | 0.298 | 3 | P07998, O00584, Q96BZ4 |
| GO:1900024 | regulation of substrate adhesion-dependent cell spreading | 0.014 | 0.298 | 3 | Q99972, P02679, P02671 |
| GO:2001237 | negative regulation of extrinsic apoptotic signaling pathway | 0.014 | 0.298 | 3 | P31749, P02671, P02679 |
| GO:0003018 | vascular process in circulatory system | 0.014 | 0.298 | 4 | P00441, P31749, P02679, P02671 |
| GO:0031224 | intrinsic component of membrane | 0.016 | 0.312 | 25 | P55286, P00742, A6NLU5, Q9Y5Y7, P13671, P07358, Q13641, Q9HAR2, Q9P2S2, O75326, Q9NPD7, P10586, P13598, Q8N3J6, Q06481, P11279, Q96BZ4, P26572, Q969P0, P07360, P54289, Q9BQT9, Q9HCU0, Q16653, P14151 |
| GO:0010770 | positive regulation of cell morphogenesis involved in differentiation | 0.018 | 0.312 | 3 | P02671, P02679, Q99972 |
| GO:1904035 | regulation of epithelial cell apoptotic process | 0.018 | 0.312 | 3 | P02671, P02679, Q9HCU0 |
| GO:0032814 | regulation of natural killer cell activation | 0.022 | 0.312 | 2 | Q96PD5, P11279 |
| GO:0033198 | response to ATP | 0.022 | 0.312 | 2 | P00441, P14151 |
| GO:0034116 | positive regulation of heterotypic cell-cell adhesion | 0.022 | 0.312 | 2 | P02671, P02679 |
| GO:0038127 | ERBB signaling pathway | 0.022 | 0.312 | 2 | P31749, Q99972 |
| GO:0045620 | negative regulation of lymphocyte differentiation | 0.022 | 0.312 | 2 | Q96PD5, Q14314 |
| GO:0046348 | amino sugar catabolic process | 0.022 | 0.312 | 2 | P26572, Q15782 |
| GO:0071276 | cellular response to cadmium ion | 0.022 | 0.312 | 2 | P31749, P00441 |
| GO:0090501 | RNA phosphodiester bond hydrolysis | 0.022 | 0.312 | 2 | P07998, O00584 |
| GO:1902042 | negative regulation of extrinsic apoptotic signaling pathway via death domain receptors | 0.022 | 0.312 | 2 | P02679, P02671 |
| GO:1902175 | regulation of oxidative stress-induced intrinsic apoptotic signaling pathway | 0.022 | 0.312 | 2 | P31749, P00441 |
| GO:2000352 | negative regulation of endothelial cell apoptotic process | 0.022 | 0.312 | 2 | P02679, P02671 |
| GO:0005758 | mitochondrial intermembrane space | 0.022 | 0.312 | 2 | P00441, Q99972 |
| GO:0004540 | ribonuclease activity | 0.022 | 0.312 | 2 | O00584, P07998 |
| GO:0005501 | retinoid binding | 0.022 | 0.312 | 2 | P05154, P07360 |
| GO:0019840 | isoprenoid binding | 0.022 | 0.312 | 2 | P07360, P05154 |
| GO:0140098 | catalytic activity, acting on RNA | 0.022 | 0.312 | 2 | P07998, O00584 |
| GO:0019835 | cytolysis | 0.023 | 0.323 | 3 | P07358, P13671, P07360 |
| GO:0002250 | adaptive immune response | 0.024 | 0.326 | 11 | A0A0B4J1V0, Q14314, P0DOX7, P0DOX5, P0DOX3, P01834, P02671, A0A0C4DH68, P07358, P13671, P07360 |
| GO:0006040 | amino sugar metabolic process | 0.032 | 0.400 | 2 | P26572, Q15782 |
| GO:0034114 | regulation of heterotypic cell-cell adhesion | 0.032 | 0.400 | 2 | P02679, P02671 |
| GO:0045580 | regulation of T cell differentiation | 0.032 | 0.400 | 2 | Q14314, P00441 |
| GO:1902041 | regulation of extrinsic apoptotic signaling pathway via death domain receptors | 0.032 | 0.400 | 2 | P02671, P02679 |
| GO:1904036 | negative regulation of epithelial cell apoptotic process | 0.032 | 0.400 | 2 | P02679, P02671 |
| GO:0004519 | endonuclease activity | 0.032 | 0.400 | 2 | P07998, O00584 |
| GO:0007422 | peripheral nervous system development | 0.034 | 0.423 | 3 | P00441, P31749, Q99972 |
| GO:1903522 | regulation of blood circulation | 0.035 | 0.433 | 4 | P31749, P02679, P54289, P02671 |
| GO:0008015 | blood circulation | 0.039 | 0.451 | 5 | P02679, P54289, P02671, P00441, P31749 |
| GO:0001701 | in utero embryonic development | 0.040 | 0.451 | 3 | P26572, P31749, P13671 |
| GO:0007283 | spermatogenesis | 0.040 | 0.451 | 3 | Q14508, P05154, P00441 |
| GO:0010769 | regulation of cell morphogenesis involved in differentiation | 0.040 | 0.451 | 3 | P02679, P02671, Q99972 |
| GO:0017157 | regulation of exocytosis | 0.040 | 0.451 | 3 | P02679, P02671, P11279 |
| GO:0048232 | male gamete generation | 0.040 | 0.4510 | 3 | P00441, Q14508, P05154 |
| GO:2001236 | regulation of extrinsic apoptotic signaling pathway | 0.040 | 0.451 | 3 | P02671, P02679, P31749 |
| GO:0003013 | circulatory system process | 0.043 | 0.455 | 5 | P31749, P00441, P54289, P02671, P02679 |
| GO:0031639 | plasminogen activation | 0.043 | 0.455 | 2 | P02679, P02671 |
| GO:0051258 | protein polymerization | 0.043 | 0.455 | 2 | P02671, P02679 |
| GO:0060047 | heart contraction | 0.043 | 0.455 | 2 | P00441, P54289 |
| GO:0002376 | immune system process | 0.043 | 0.455 | 23 | Q96BZ4, P31749, P00441, P07360, P13598, P0DOX5, P11279, P02671, Q96PD5, Q9HCU0, P0DOX7, P14151, Q16653, A0A0B4J1V0, Q14314, O00584, P07358, P13671, P0DOX3, A0A0C4DH68, P01834, O75326, P07998 |
| GO:2001233 | regulation of apoptotic signaling pathway | 0.045 | 0.463 | 4 | P02679, P02671, P00441, P31749 |
| GO:0005887 | integral component of plasma membrane | 0.046 | 0.463 | 12 | P07360, P07358, P13671, Q9Y5Y7, P11279, P13598, P14151, P10586, O75326, Q9BQT9, Q13641, Q9HAR2 |
| GO:0001817 | regulation of cytokine production | 0.047 | 0.463 | 7 | Q96BZ4, Q96PD5, O75326, P00441, Q16653, Q9BXJ3, P0DOX3 |
| GO:0001649 | osteoblast differentiation | 0.047 | 0.463 | 3 | Q99972, O75326, P31749 |
| GO:0072376 | protein activation cascade | 0.047 | 0.463 | 3 | P02671, P00742, P02679 |
| GO:0072378 | blood coagulation, fibrin clot formation | 0.047 | 0.463 | 3 | P02679, P00742, P02671 |

**S****upplementary Table S6** GO enrichment analysis of the serum identified proteins.（Fold change=1.5）

| GO_ID | Category | *p*-value | Corrected *p*-value | No. | Gene |
| --- | --- | --- | --- | --- | --- |
| GO:0060042 | retina morphogenesis in camera-type eye | 0.005 | 0.444 | 2 | P02753, P54802 |
| GO:0048593 | camera-type eye morphogenesis | 0.007 | 0.444 | 2 | P02753, P54802 |
| GO:0071634 | regulation of transforming growth factor beta production | 0.007 | 0.444 | 2 | P02751, Q14766 |
| GO:0050431 | transforming growth factor beta binding | 0.007 | 0.444 | 2 | Q14766, Q9NPY3 |
| GO:0072359 | circulatory system development | 0.009 | 0.444 | 5 | P39060, P02751, P33151, Q14766, P02753 |
| GO:0060041 | retina development in camera-type eye | 0.010 | 0.444 | 2 | P54802, P02753 |
| GO:0048598 | embryonic morphogenesis | 0.012 | 0.444 | 3 | P02751, P02753, P54802 |
| GO:0003205 | cardiac chamber development | 0.013 | 0.444 | 2 | Q14766, P02753 |
| GO:0048592 | eye morphogenesis | 0.013 | 0.444 | 2 | P02753, P54802 |
| GO:0048562 | embryonic organ morphogenesis | 0.017 | 0.444 | 2 | P54802, P02753 |
| GO:0090596 | sensory organ morphogenesis | 0.017 | 0.444 | 2 | P54802, P02753 |
| GO:0043687 | post-translational protein modification | 0.019 | 0.444 | 4 | Q9BTY2, P02751, Q14766, Q8NBP7 |
| GO:0007601 | visual perception | 0.021 | 0.444 | 2 | P02753, P39060 |
| GO:0032091 | negative regulation of protein binding | 0.021 | 0.444 | 2 | Q03591, Q8NBP7 |
| GO:0050953 | sensory perception of light stimulus | 0.021 | 0.444 | 2 | P02753, P39060 |
| GO:0001568 | blood vessel development | 0.022 | 0.444 | 4 | P39060, P33151, P02751, Q14766 |
| GO:0001944 | vasculature development | 0.022 | 0.444 | 4 | P39060, P33151, P02751, Q14766 |
| GO:0070013 | intracellular organelle lumen | 0.026 | 0.444 | 8 | P33151, Q8NBP7, Q14766, P02751, P39060, P54802, Q9BTY2, P19652 |
| GO:0005788 | endoplasmic reticulum lumen | 0.029 | 0.444 | 5 | Q9BTY2, Q14766, Q8NBP7, P02751, P39060 |
| GO:0007178 | transmembrane receptor protein serine/threonine kinase signaling pathway | 0.030 | 0.444 | 2 | Q14766, P33151 |
| GO:0051100 | negative regulation of binding | 0.030 | 0.444 | 2 | Q03591, Q8NBP7 |
| GO:0005775 | vacuolar lumen | 0.035 | 0.444 | 3 | Q9BTY2, P19652, P54802 |
| GO:0019897 | extrinsic component of plasma membrane | 0.035 | 0.444 | 2 | Q8NBP7, P33151 |
| GO:0004553 | hydrolase activity, hydrolyzing O-glycosyl compounds | 0.035 | 0.444 | 2 | Q9BTY2, P54802 |
| GO:0019955 | cytokine binding | 0.035 | 0.444 | 2 | Q14766, Q9NPY3 |
| GO:0044403 | biological process involved in symbiotic interaction | 0.039 | 0.444 | 4 | P02751, P08603, Q03591, Q9NPY3 |
| GO:0043010 | camera-type eye development | 0.040 | 0.444 | 2 | P54802, P02753 |
| GO:0048568 | embryonic organ development | 0.040 | 0.444 | 2 | P02753, P54802 |
| GO:0000323 | lytic vacuole | 0.041 | 0.444 | 4 | Q9BTY2, P19652, Q8NBP7, P54802 |
| GO:0005764 | lysosome | 0.041 | 0.444 | 4 | Q8NBP7, P54802, Q9BTY2, P19652 |
| GO:0005773 | vacuole | 0.044 | 0.444 | 4 | P54802, Q8NBP7, P19652, Q9BTY2 |
| GO:0001654 | eye development | 0.046 | 0.444 | 2 | P54802, P02753 |
| GO:1901136 | carbohydrate derivative catabolic process | 0.046 | 0.444 | 2 | P54802, Q9BTY2 |
| GO:0016798 | hydrolase activity, acting on glycosyl bonds | 0.046 | 0.444 | 2 | P54802, Q9BTY2 |
| GO:0009790 | embryo development | 0.047 | 0.444 | 3 | P02751, P02753, P54802 |

**Supplementary Table S7** KEGG enrichment analysis of the CSF identified proteins.（Fold change=1.5）

| Term | Database | ID | Input number | Background number | *p*-value | Corrected *p*-value | protein info |
| --- | --- | --- | --- | --- | --- | --- | --- |
| Complement and coagulation cascades | KEGG PATHWAY | ko04610 | 7 | 55 | 0.00365 | 0.03759 | P13671;P07360;P02671;P07358;P05154;P02679;P00742 |
| Insulin resistance | KEGG PATHWAY | ko04931 | 2 | 4 | 0.00898 | 0.03759 | P10586;P31749 |
| Prion disease | KEGG PATHWAY | ko05020 | 4 | 22 | 0.00909 | 0.03759 | P00441;P13671;P07360;P07358 |
| Platelet activation | KEGG PATHWAY | ko04611 | 3 | 12 | 0.01002 | 0.03759 | P31749;P02671;P02679 |
| Longevity regulating pathway - multiple species | KEGG PATHWAY | ko04213 | 2 | 5 | 0.01460 | 0.04381 | P00441;P31749 |
| Insulin signaling pathway | KEGG PATHWAY | ko04910 | 2 | 6 | 0.02137 | 0.05343 | P10586;P31749 |
| N-Glycan biosynthesis | KEGG PATHWAY | ko00510 | 2 | 7 | 0.02919 | 0.06256 | P26572;Q14697 |
| Systemic lupus erythematosus | KEGG PATHWAY | ko05322 | 3 | 19 | 0.03672 | 0.06330 | P13671;P07360;P07358 |
| Autophagy - animal | KEGG PATHWAY | ko04140 | 2 | 8 | 0.03798 | 0.06330 | P31749;P11279 |
